# Transcriptional profiling of milk fat synthesis in mammary gland of dairy cows with different forage quality using RNA sequencing

**DOI:** 10.3389/fvets.2025.1469169

**Published:** 2025-03-26

**Authors:** Hang Zhang, Changjin Ao, Khas-Erdene, Ni Dan

**Affiliations:** ^1^College of Animal Science and Technology, Inner Mongolia Minzu University, Tong Liao, Inner Mongolia, China; ^2^College of Animal Science, Inner Mongolia Agricultural University, Hohhot, Inner Mongolia, China; ^3^College of Life Science, Inner Mongolia Minzu University, Tong Liao, Inner Mongolia, China

**Keywords:** dairy cow, forage quality, lipid metabolic, milk fat, RNA sequencing

## Abstract

**Introduction:**

This study was designed to determine the effects of diets containing different-quality forages and concentrate content on milk composition and lipogenic enzyme expression in the mammary glands of dairy cows using RNA sequencing.

**Methods:**

Thirty Holstein cows were randomly assigned to one of three treatments: mixed forage consisted of hay, silage, and alfalfa forage to a concentrate ratio of 54:46 (MF); corn stover as forage and forage to a concentrate ratio of 35:65 (CSA); corn stover as forage and forage to a concentrate ratio the same as MF (CSB). Mammary tissue biopsies were performed to analyze lipogenic enzyme expression using RNA-seq.

**Results:**

Treatments did not affect dry matter intake, milk protein, or lactose. The milk yield, fat content and saturated fatty acids (SFAs), and short- and medium-chain fatty acids (SMFAs) contents were lower in CSA and CSB than in MF. Still, the unsaturated FA and long-chain FA contents were higher than in MF. We used RNA-seq to compare analyses of three groups of mammary tissue in transcriptomics, Gene Ontology and KEGG-enriched pathways. Differentially expressed genes (DEGs) involved in lipid metabolic pathways and the FA biosynthesis pathway in MF were significantly higher than in CSB. In contrast, DEGs of FA extension and unsaturated FA synthesis pathway were significantly lower than in CSB.

**Conclusion:**

Corn stover as a forage diet reduced the milk yield, fat content, SMFAs, SFAs, and the gene expression of mammary lipogenic enzymes in dairy cows.

## 1 Introduction

Milk yield and its components are two of the most important economic traits influenced by nutrition, environmental factors, cow breed, cow age, and seasonality (1). Milk fat is a part of milk components that is a great concern for consumers (2), and its modulation through diet has been researched in many studies. Milk fat is the milk component most susceptible to diet manipulation in cows (3). Diets with different qualities of forages had distinct effects on milk fatty acid (FA) composition, being related to the expression of lipid metabolism-related genes in the milk FA composition. Therefore, diet may be key to milk FA-regulating mechanisms (4).

Recently, Chinese wildrye and alfalfa hay have been used as the main forages for feeding dairy cows in China. However, alfalfa hay is of high nutrition but in short supply. Large amounts of corn stover are produced yearly (5). Still, the low nutrient level in corn stover might result in lower livestock production if it is used as the main forage source. A diet with Chinese wildrye and alfalfa hay as the main forages increased the milk yield, milk fat and protein contents, and the expression of mammary lipogenic enzymes compared with corn stover as the forage fed to ruminants (6, 7). Low-quality forage often requires more concentrate to maintain the nutrition level of the diet. Applying a high ratio of concentrate in dairy cow diets may decrease milk fat content, especially when the concentrates contain a large amount of starch (8). Feeding different-quality diets also affects the mRNA abundance of milk lipid metabolism-related genes in the mammary glands of dairy cows (9, 10). Milk fat synthesis is a complex procedure involving a wide gene network with ambiguous regulation (11).

Along with the rapid development and cost reduction of next-generation sequencing, sequence-based assays of transcriptome RNA-seq have recently become a comprehensive and accurate tool for gene expression pattern analyses. RNA-seq enables analysis of the complexity of whole eukaryotic transcriptomes with less bias, greater dynamic range, lower frequency of false-positive signals, and higher reproducibility than microarray technology (12, 13). Several studies on bovine milk transcriptome have used RNA-seq techniques (14–16). However, no studies have been published on how different forage diets affect the bovine mammary gland transcriptome using RNA-seq. The present study aimed to identify genes and pathways involved in diets composed of different-quality forages on mammary lipogenic enzymes in mid-lactating dairy cows using RNA-seq technology. Comprehensive information on the molecular events of diet-affected milk fat will enhance our understanding of milk composition and lead to the identification of novel genes or transcripts that regulate milk fat.

## 2 Materials and methods

### 2.1 Animals and sample collection

This study was conducted in accordance with the guidelines set forth by the Animal Care and Ethics Committee of Inner Mongolia Agricultural University (NO. 2023016). A total of 30 multiparous Holstein dairy cows (body weight = 554 ± 21 kg, days in milk = 120 ± 24 d) were randomly assigned to one of three groups (10 cows per group) with a 60-day experimental period. The last three experimental days were those during which the sample collection took place. Cows were housed in individual stalls with free access to drinking water, fed TMR *ad libitum*, and milked twice daily at 06:00 and 18:00 h during this trial. Three TMRs with different forages and concentrate-to-forage ratios were designed. The diets (on DM base) were: [1] mixed forages including hay (4%), corn silage (27%), and alfalfa (23%) with 46% of concentrate (MF); [2] corn stover as forage formulated similar proximate nutrient levels with MF, and forage to a concentrate ratio of 35:65 (CSA); and [3] 54% of corn stover and 46% of concentrate (CSB).

The feed intake for each cow was measured daily and adjusted using the DM of TMR to determine the daily DM intake (DMI). Samples of TMR and orts were collected for three consecutive days and stored at −20°C before analysis of the nutrient components of diets (Tables 1, 2). The milk yield was recorded daily throughout the experiment. Milk samples were collected during the sampling period of the last 3 days. The samples were mixed thoroughly and stored at 4°C with preservatives until analysis of milk fat, protein, and lactose using MilkoScan^TM^ Minor Type 78110 (FOSS Analytical A/S 69, DK 3400, Hillerod, Denmark) and somatic cell count (SCC) using ADAM SCC II (Nanoentek Inc., Seoul, Korea).

**Table 1:** Ingredients and composition of the experimental diets (% of dry matter).

**Item**	**Diets^†^**		
	**CSA**	**CSB**	**MF**
Hay	0	0	3.7
Corn silage	0	0	26.7
Alfalfa hay	0	0	23.4
Cornstalk	35	53.8	0
Ground corn grain	34.61	24.6	24.6
Soybean meal, 49.0% CP	20.82	14.8	14.8
Whole cottonseed	7.18	5.1	5.1
Calcium bicarbonate	0.84	0.6	0.6
Edible salt	0.7	0.5	0.5
Mineral-vitamin mix^‡^	0.84	0.6	0.6
Total	100	100	100

† MF, with hay, corn silage, and alfalfa mixed as roughage; CSA, with only corn stalk as roughage and with the same level of proximate nutrients to MF; CSB, with only corn stalk as roughage and with the same proportion of roughage to MF.

‡ Formulated to provide (per kilogram of DM) 500,000–700,000 IU of vitamin A, 110,000–120,000 IU of vitamin D3, 8,000–10,000 IU of vitamin E, 7,000–10,000 mg of Zn, 40–80 mg of Se, 84 mg of I, 1,400–1,750 mg of Fe, 30–40 mg of Co, 1,400–3,500 mg of Mn, and 1,400–1,600 mg of Cu.

**Table 2:** Chemical composition and fatty acid composition of the experimental diets.

	**Diets^†^**		
**Item**	**CSA**	**CSB**	**MF**
**Chemical composition**^‡^ **(% of DM)**			
CP	18.38	13.61	18.14
EE	4.10	2.84	3.97
NDF	33.10	44.30	32.30
ADF	20.20	29.10	21.30
Ca	0.82	1.06	0.82
P	0.40	0.46	0.39
ASH	7.39	7.93	7.47
Starch	25.39	15.32	21.50
NEL (Mcal kg^−1^)	1.58	1.04	1.57
**Fatty acid (g/100 g of total fatty acids)**			
C16:0	21.22	19.98	24.35
C16:1	0.42	0.51	0.62
C18:0	3.38	3.38	3.01
C18:1c9	23.1	24.58	21.73
C18:2n6	44.8	43.69	44.16
C18:3n3	4.75	3.92	2.55
Others	2.33	3.94	3.58

† MF, with hay, corn silage, and alfalfa mixed as roughage; CSA, with only corn stalk as roughage and with the same level of proximate nutrients to MF; CSB, with only corn stalk as roughage and with the same proportion of roughage to MF.

‡ Compositions of the experimental diets were calculated according to the chemical analysis and inclusion rate of ingredients, as indicated in Table 1. DM, dry matter; CP, crude protein; EE, crude fat (ether extract); ADF, acid detergent fiber; NDF, neutral detergent fiber; NEL, net energy for lactation; The composition of CP, EE, NDF, ADF, Ca, P, ASH calculated the chemical analysis based on the Ministry of Agriculture of P.R. China (2004); Other composition calculated by near-infrared spectroscopy (FOSS NIRS DS 250).

Milk FAs were extracted using a previously described procedure (6). FA methyl esters from milk and feed samples were analyzed using a Shimadzu GC 2014 system (Shimadzu, Kyoto, Japan) gas chromatography equipped with a 100 m HP 88 fused silica capillary column × 0.25 mm i.d., coated with 0.2 μm film thickness (Agilent J&W, Santa Clara, CA, USA).

### 2.2 Mammary tissue sampling and RNA isolation

Five cows from each treatment were randomly selected to perform mammary tissue biopsies after milking on the last day of the experiment. The biopsy was conducted according to a previously described procedure (17). The mammary tissue biopsies (~500 mg/animal) were rinsed in a 0.9% sterile saline solution and then frozen in liquid nitrogen. The samples were kept at −80°C until RNA extraction.

Total RNA was extracted from each frozen mammary biopsy using an RNA Prep Pure Tissue kit (Tiangen Biotech Co., Ltd., Beijing, China). The concentration and purity of isolated total RNA were determined using a BioTek Synergy H4 Hybrid Reader (BioTek Laboratory Instrument, Winooski, VT, USA). The mRNA integrity was evaluated by observing 18 S and 28 S ribosomal bands after agarose gel electrophoresis. Each tissue was performed in five replicates.

### 2.3 Transcriptome sample preparation and library construction

A total of 30 μg of RNA per cow was used as the input material for the RNA sample preparations. Sequencing libraries were generated using Solexa on the Illumina platform. The library construction procedure was conducted according to the method by Cui et al. (18). Oligo(dT)_25_ magnetic beads purified mRNA from total RNA. Then, divalent cations under 94°C denaturation in RNA fragmentation buffer fragmented the mRNAs. Random oligonucleotides were used to synthesize first-strand cDNA. RNase H and DNA polymerase I subsequently generated second-strand cDNA. After adenylation of the 3′ ends of DNA fragments, adapter oligonucleotides were ligated to prepare for gel purification recovery using a Gel Extraction Kit (Illumina, CA). The PCR primer selectively enriched DNA fragments with ligated adaptor molecules on both ends in a 12-cycle PCR reaction. Gel Extraction Kit QIA quick Gel and Mini Columm were used for gel purification and recovery and the extraction of DNA fragments of ~200 bp in length. The purified products were quantified using an Agilent high-sensitivity DNA assay on the Agilent 2100 Bioanalyzer system. Illumina sample clusters were prepared using Cluster Station according to the manufacturer's instructions. After cluster generation, an Illumina Hiseq 2000 platform sequenced the libraries.

### 2.4 Quality control for paired-end reads and *de novo* splicing

A large amount of sample data was obtained using Solexa RNA paired-end sequencing. Given the impact of the Solexa data error rate on the results, quality preprocessing of the original data was performed. The sliding window method removed low-quality fragments: the quality threshold was 20 (error rate = 1%), the window size was 5 bp, and the length threshold was 35 bp. The length threshold of the sequence containing part N in the reads was 35 bp. After preprocessing, we obtained 121 million effective RNA-seq data. The data volume was 10 G, and the average length was 86.65 bp. The amount of sample sequencing data was ~5 G.

Effective reads of the three samples were combined for *de novo* splicing. The software Trinity, version trinityrnaseq_r2012-10-05, and the paired-end splicing method were used. Repeat the splicing sequence, and finally get 199,039 transcripts with a length >200 bp and a size of 216 Mb. Taking the longest transcript under each Loci (comp^*^_c^*^_) as Unigene (the software's Chrysalisclusters module), we obtained 148,900 unigenes, 110 Mb in size. The sample unigene was compared with the public data gene, and function annotation was conducted using the similarity of the gene. Annotations with a similarity >30% and e < 1e^−5^ were obtained, and all annotation details obtained were merged and compared with NR, SWISS-PROT, TrEMBL, Cdd, pfam, and eukaryotic complete genomes (COG) libraries. The unigene sample was compared with the public data gene, and function annotation was conducted using the similarity of genes, including NR, SWISS-PROT, TrEMBL, Cdd, pfam, COG classification, Gene Ontology (GO), and KEGG annotations. Annotation, differentially expressed genes (DEGs) analysis, and enrichment analysis were performed only for genes with a length ≥500 bp. GO, COG cluster analysis, and KEGG metabolic pathway analysis were conducted by comparing the predicted proteins in the mammary tissue of dairy cows. These analytical methods helped predict the effects of different roughage qualities on the functions of genes related to milk fat synthesis in the mammary glands of dairy cows from the overall transcriptome level.

### 2.5 Validation of RNA-seq data using RT-qpcr

qRT-PCR was performed on nine randomly selected differentially expressed milk fat synthesis-related genes using the total RNA used for RNA-seq to validate the repeatability and reproducibility of the gene expression data obtained using RNA sequencing in Holstein cows. RNA was converted into cDNA by using 2 μL of 100 ng total RNA, 2 μL of 5 × PrimeScript RT Master Mix, and 6 μL of DNase/RNase free water (TaKaRa, Tokyo, Japan) at 37°C for 15 min and then 85°C for 5 s. RT-qPCR was performed using 2 μL of diluted cDNA combined with 10 μL of 2 × SYBR premix Ex Taq II (TaKaRa, Tokyo, Japan), 0.4 μL each of 10 μM forward and reverse primers, and 7.2 μL DNase/RNase free water in 96 well microwell plates of the BIO-RAD IQ5 Multicolor RT-qPCR Detection System (USA). RT-qPCR reactions were performed 30 s with pre-denaturation at 95°C, followed by 40 cycles of 15 s denaturation at 95°C, 20 s annealing at 58°C and 30 s extension at 72°C. RT-qPCR analysis was performed using the 2^−ΔΔCT^ method with β-actin as the reference gene. The primer sequences and annealing temperature are summarized in Table 3. The primers were designed according to Zhang et al. (17) and were synthesized by Shanghai Sangon Biological Engineering and Technology Service Co. Ltd. (Shanghai, China).

**Table 3:** Polymerase chain reaction amplification primer sequence and annealing of genes.

**Gene**	**Accession**	**Primer sequences**	**Tm (°C)**
β-Actin	NM_173979.3	F. 5′-AACTCCATCATGAAGTGTGACG	59
		R. 5′- GATCCACATCTGCTGGAAGG	
SCD	AY241933	F. 5′- TCCTGTTGTTGTGCTTCATCC	58
		R.5′- GGCATAACGGAATAAGGTGGC	
ACACA	AJ132890	F. 5′- CATCTTGTCCGAAACGTCGAT	58
		R. 5′- CCCTTCGAACATACACCTCCA	
FASN	CR552737	F. 5′-ACCTCGTGAAGGCTGTGACTCA	58
		R. 5′-TGAGTCGAGGCCAAGGTCTGAA	
LPL	BC118091	F. 5′- ACACAGCTGAGGACACTTGCC	60
		R. 5′- GCCATGGATCACCACAAAGG	
PPARA	BT020756	F. 5′-CGGTGTCCACGCATGTGA R. 5′-TCAGCCGAATCGTTCTCCTAAA	59
CD36	BC103112	F. 5′-CCTCTTGGCAACCACTTTCA R. 5′-GCAATGAGCCCACAGTTTCG	62
FABP3	DN518905	F. 5′-GAACTCGACTCCCAGCTTGAA R. 5′-AAGCCTACCACAATCATCGAAG	59
ACSL1	BC119914	F. 5′-GTGGGCTCCTTTGAAGAACTGT R. 5′-ATAGATGCCTTTGACCTGTTCAAAT	57
LPIN1	DV797268	F. 5′- TGGCCACCAGAATAAAGCATG R. 5′- GCTGACGCTGGACAACAGG	59

### 2.6 Calculation and statistical analysis

DMI and milk performance were analyzed using a two-sided Student's *t*-test with the SAS 9.0 software package (SAS Inst. Inc., Cary, NC, USA). A 95% confidence interval was used in all cases as the default for the hypothesis test. The assumptions of normality were validated using the univariate procedure, and the results were deemed significant when *P* < 0.05. Differential expression genes (DEGs) were identified using value of logarithmic transformed fold-change [log(FC)] ≥ 1 or < -1.

## 3 Results

### 3.1 Feed intake and milk production performance

There were no differences in DMI between the dietary treatments (Table 4). The milk yield was lower in cows fed the CSB diet, and milk fat content was lower in cows fed the CSA diet than in cows fed the MF diet (*P* < 0.05). Milk protein and lactose contents were not affected after cows were fed different diets. SCC was not affected by diet and remained within the normal range. Compared with cows fed the MF diet, saturated FAs (SFAs) and short- and medium-chain FAs (SMFAs) in milk were lower (*P* < 0.05) in cows fed the CSA and CSB diet, whereas unsaturated FA (UFA) and long-chain FAs (LCFAs) in milk fat were higher (*P* < 0.05).

**Table 4:** The effects of dietary treatments on milk performance.

**Item**	**Treatment^†^**				
	**CSA**	**CSB**	**MF**	**SEM**	***P***
Milk yield, kg/d	23.21^b^	17.41^c^	26.41^a^	1.51	<0.01
DMI^‡^, kg/d	16.12	15.83	16.42	0.42	0.58
Fat, %	3.71^b^	4.03^ab^	4.26^a^	0.15	0.04
Protein, %	3.10	3.00	3.20	0.05	0.11
Lactose, %	4.71	4.54	4.78	0.08	0.67
SCC^§^ (^*^1,000 mL^−1^)	25.24	29.87	24.67	6.59	0.35
FA^¶^, g/100 g of FA SFA	64.24^b^	63.24^b^	71.98^a^	1.86	0.01
UFA	35.76^a^	36.76^a^	27.02^b^	1.34	<0.01
SMFA	19.86^b^	20.63^b^	22.32^a^	1.68	0.04
LCFA	80.14^a^	79.37^a^	77.68^b^	1.21	0.04

† MF, with hay, corn silage, and alfalfa mixed as roughage; CSA, with only corn stalk as roughage and with the same level of proximate nutrients to MF; CSB, with only corn stalk as roughage and with the same proportion of roughage to MF.

‡ DMI, dry matter intake.

§ SCC, somatic cell count.

¶ SFA, saturated fatty acids; UFA, unsaturated fatty acids; PUFA, polyunsaturated fatty acids; SMFA, short- and medium-chain fatty acids (C < 16); LCFA, long-chain fatty acids (C ≥ 16).

^a, b, c^Means with the different letters within a row are significantly different (*P* < 0.05).

### 3.2 Sequencing of the bovine mammary gland transcriptome

**Figure 1:**
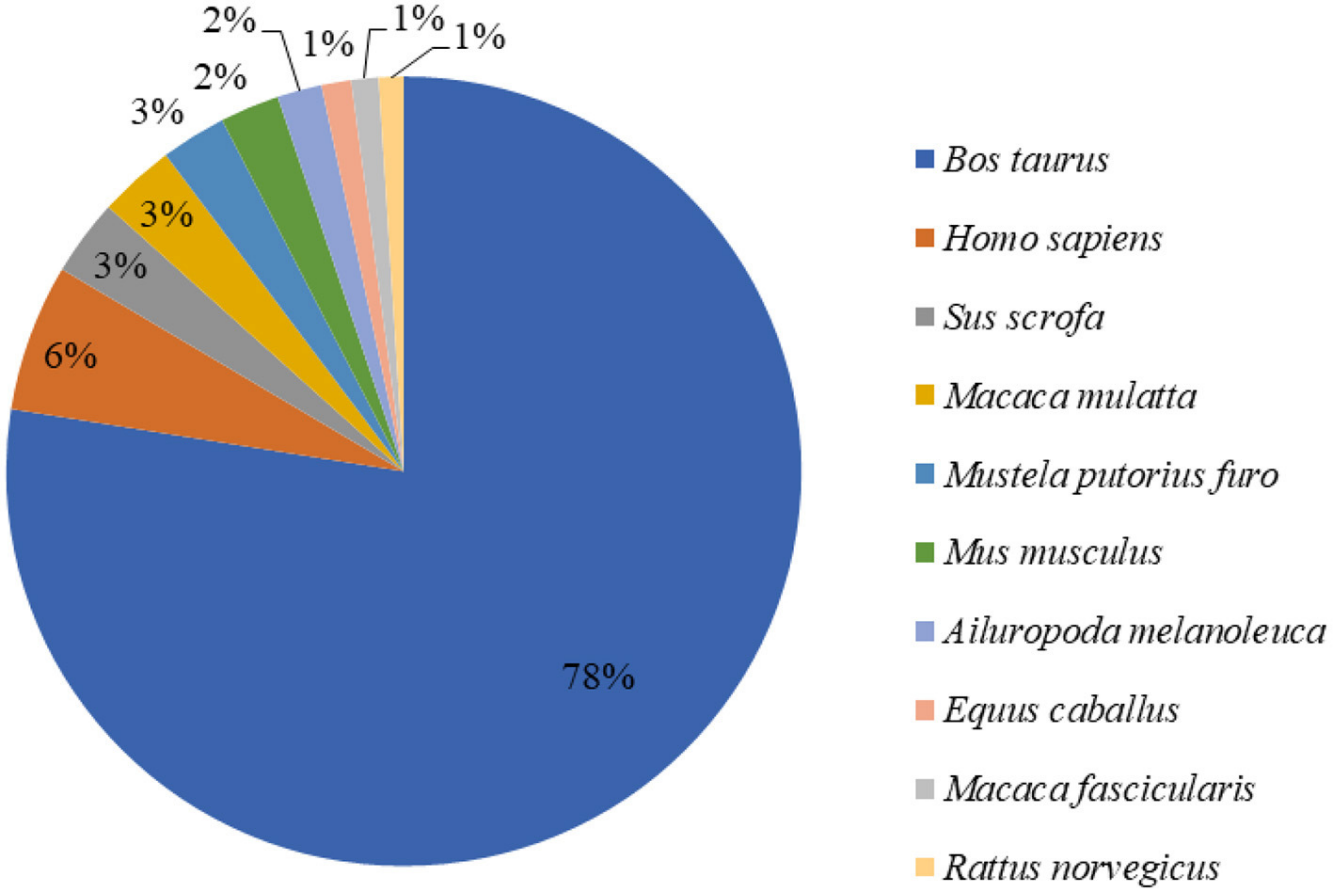
Nr tax statistics for mammary gland tissue of dairy cows.

We sequenced cDNA libraries of three mammary gland samples from 15 Holstein cows with CSA, CSB, and MF diets. We acquired 49,969,014–54,513,152 paired-end reads of 86.65 bp in length per sample. As a result, the total read length was 10 gigabases (Gb) for the three samples. The sample unigene was compared with the public data gene, and function annotation was performed using gene similarity. The similarity with the NR database was 30.35%. Among the 148,900 unigenes obtained in NR annotations, 91.45% were the 10 species with the largest number of Unigene annotations, and 78% of unigenes matched the protein sequence of *Bos taurus* (Figure 1), which also verified the reliability of our sequencing data (17).

### 3.3 COG, GO, and KEGG functional classification analysis

**Figure 2:**
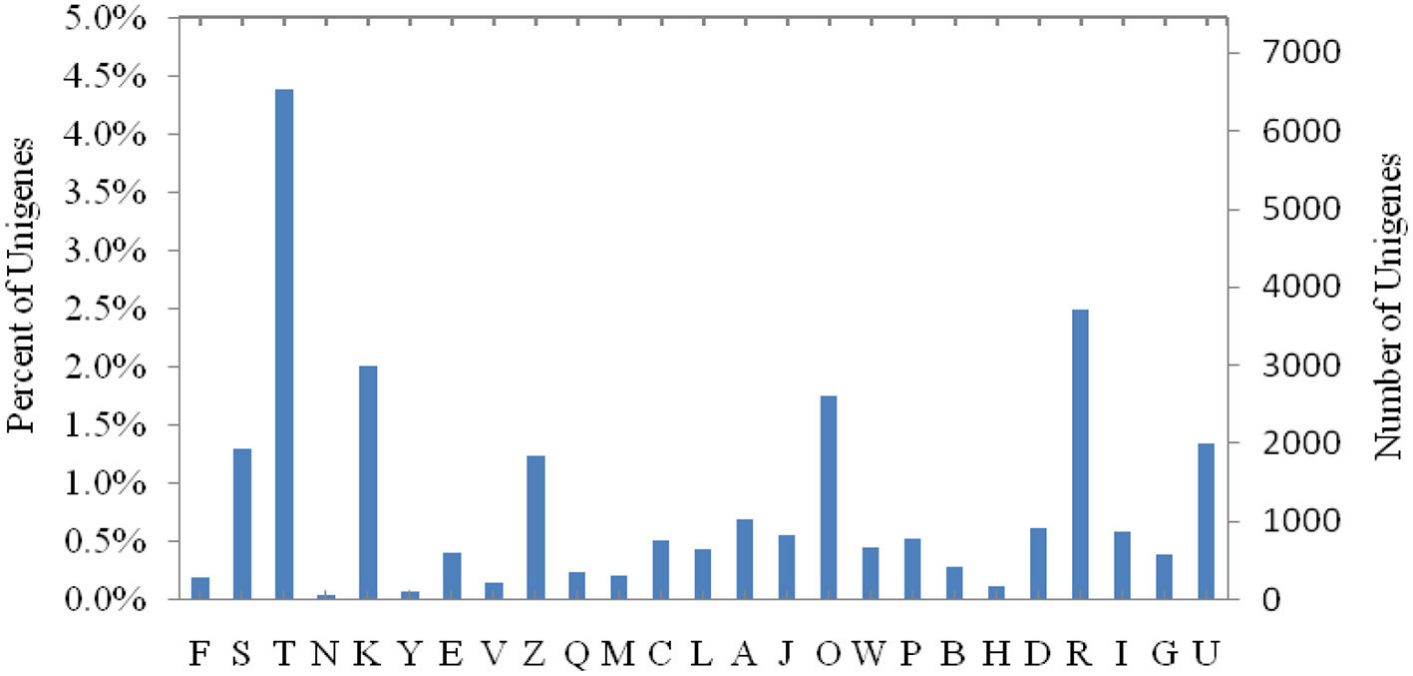
COG functional categories. [A] RNA processing and modification. [B] Chromatin structure and dynamics. [C] Energy production and conversion. [D] Cell cycle control, cell division, and chromosome partitioning. [E] Amino acid transport and metabolism. [F] Nucleotide transport and metabolism. [G] Carbohydrate transport and metabolism. [H] Coenzyme transport and metabolism. [I] Lipid transport and metabolism. [J] Translation, ribosomal structure, and biogenesis. [K] Transcription. [L] Replication, recombination, and repair. [M] Cell wall/membrane/envelope biogenesis. [N] Cell motility. [O] Posttranslational modification, protein turnover, and chaperones. [P] Inorganic ion transport and metabolism. [Q] Secondary metabolite biosynthesis, transport, and catabolism. [R] General function prediction only. [S] Function unknown. [T] Signal transduction mechanisms. [U] Intracellular trafficking, secretion, and vesicular transport. [V] Defense mechanisms. [W] Extracellular structures. [Y] Nuclear structure. [Z] Cytoskeleton.

Clusters of orthologous groups for COG functional classification prediction of genes found that 18,828 unigenes were annotated into 25 COG classifications. Among them, the largest number of unigenes, about 6,518, were related to the function of signal transduction mechanisms, and 877 unigenes were related to lipid transport and metabolic functions (Figure 2). There were 469 gene expressions that significant differed in the fat transcript and metabolism between treatments (Table 5).

**Table 5:** Difference genes of unigene in fat transport and metabolism analyzed by COG.

	**FA- FC.p (up)^†^**	**FA-FC.n (down)^†^**
CSA_VS _CSB^‡^	151	16
CSA_VS _MF^‡^	39	65
CSB_VS _MF^‡^	33	398

† FC. p, fold. Change. Positive (value≥ 1); FC. n, fold. Change. Negative (value < -1).

‡ MF, with hay, corn silage, and alfalfa mixed as forage; CSA, with only corn stalk as forage and with the same level of proximate nutrients to MF; CSB, with only corn stalk as forage and with the same proportion of forage to MF.

**Figure 3:**
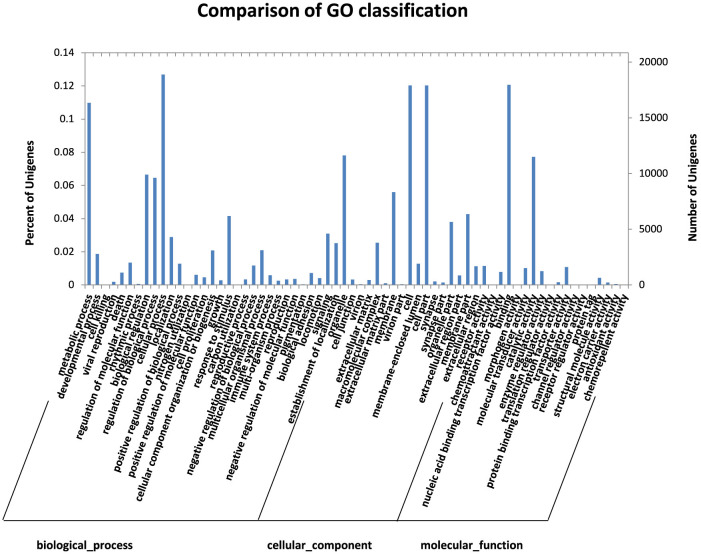
Comparison of GO classifications.

The results of unigene based on blast-uniprot (i.e., combined with SWISS-PROT and TrEMBL results) were obtained, and the obtained uniprot number was used to compare the GO term. The GO function classification and prediction of unigene showed that 211,947 unigenes were classified as GO, of which 96,295 were classified as biological processes and 77,729 as cellular components. A total of 37,950 were classified as molecular functions. The functions of the sample genes clustered in the cellular and metabolic processes at 18,899 and 16,358, respectively, in the biological process classification. There were 680 differentially expressed functions in the biological process among the samples. The cellular component was concentrated in the cell and cell parts, and 149 differentially expressed proteins were found in the cellular component among the three samples. In the classification of molecular functions, 17,970 binding and 11,509 catalytic activities were concentrated in the catalytic activities. The samples had 350 differentially expressed molecular functions (Figure 3). The differentially expressed genes of the samples were primarily involved in biological processes, cell components, and molecular functions, and the term related to membrane composition was the most significantly enriched GO. Second, the differentially expressed genes were significantly correlated with biological functions integral to the membrane, intracellular membrane, transport, and plasma membrane.

KEGG pathway analysis of the genes revealed the metabolic pathways involved in Unigene in the mammary glands of dairy cows and the functions of genes. A total of 23,095 transcripts were annotated, with 1,091 enzymes involved in 302 metabolic pathways. The number of transcripts involved in the cancer pathway was the highest, with 862, followed by the MAPK signaling pathway, with 674, and Focal adhesion reached 663. The most significantly different genes among the groups were in the cancer pathway. Different dietary patterns significantly affected milk fat and FA composition. Therefore, we summarized the milk fat anabolic pathways. There were 935 transcripts obtained from all transcript libraries that participated in 16 lipid metabolism pathways (Table 6).

**Table 6:** Classification of lipid metabolism pathways and numbers of unigenes.

**Pathway ID**	**Pathway name**	**Unigene number**
ko00564	Glycerophospholipid metabolism	204
ko00561	Glycerolipid metabolism	111
ko00590	Arachidonic acid metabolism	108
ko00600	Sphingolipid metabolism	83
ko00071	Fatty acid metabolism	82
ko00565	Ether lipid metabolism	55
ko01040	Biosynthesis of unsaturated fatty acids	45
ko00062	Fatty acid elongation	44
ko00140	Steroid hormone biosynthesis	38
ko00120	Primary bile acid biosynthesis	38
ko00591	Linoleic acid metabolism	32
ko00592	alpha-Linolenic acid metabolism	30
ko00100	Steroid biosynthesis	29
ko00072	Synthesis and degradation of ketone bodies	14
ko00061	Fatty acid biosynthesis	11
ko00073	Cutin, suberine, and wax biosynthesis	11

### 3.4 DEGs in the mammary gland of dairy cows with different qualities diets

**Figure 4:**
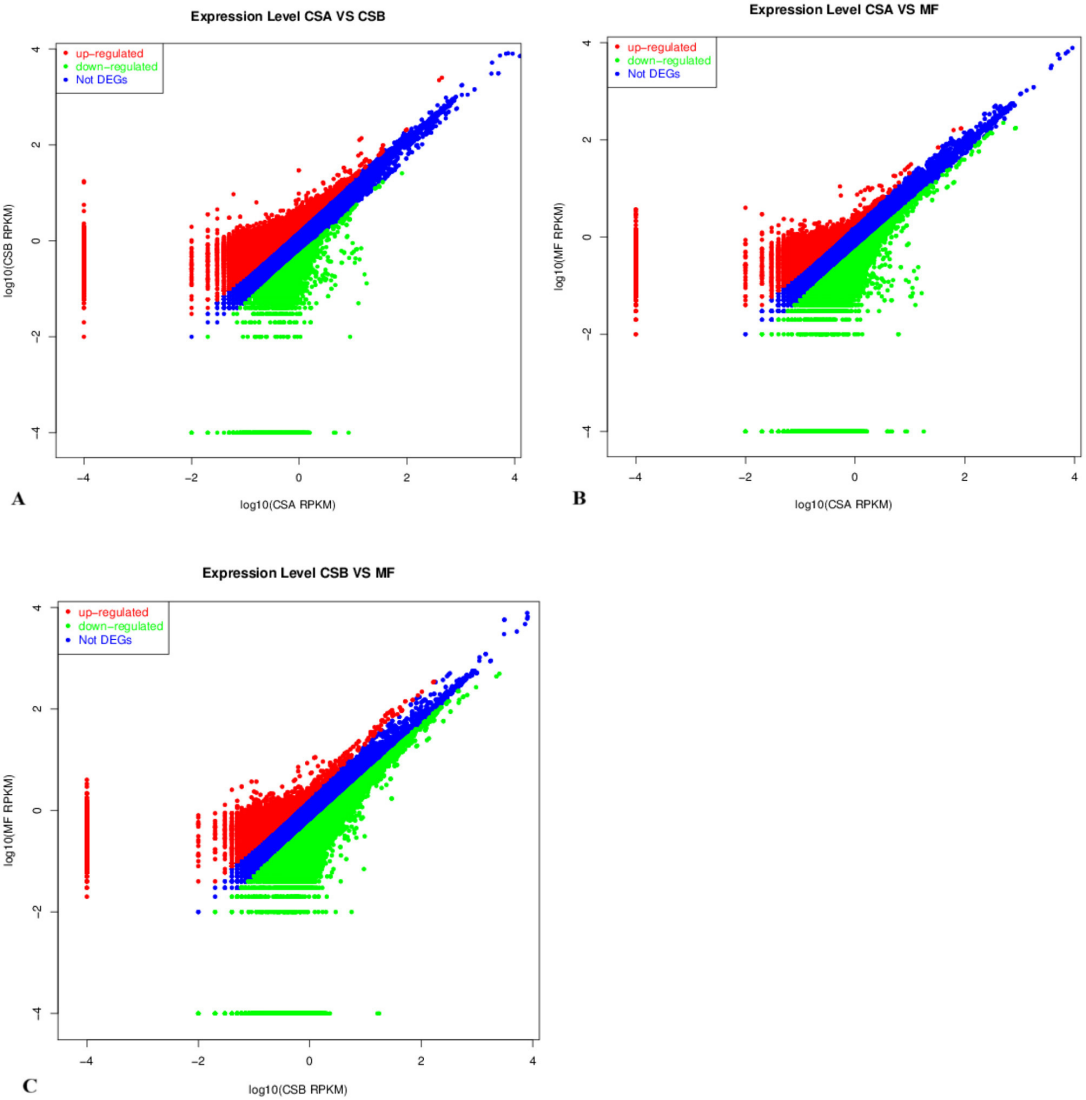
The DEGs in the mammary tissue of cows with different roughage diets. **(A)** DEGs identified in the mammary tissue between CSA_VS_CSB. **(B)** DEGs identified in the mammary tissue between CSA_VS_MF. **(C)** DEGs identified in the mammary tissue between CSB_VS_MF. The red and green dots represent the up-regulated and down-regulated gene (*P* ≤ 0.01), respectively; the blue dots represent the transcripts whose expression levels did not reach statistical significance (*P* > 0.01). MF, with hay, corn silage, and alfalfa mixed as forage; CSA, with only corn stalk as forage and with the same level of proximate nutrients to MF; CSB, with only corn stalk as forage and with the same proportion of forage to MF.

The expression abundance values of sample transcripts were used for differential expression analysis, including fold change analysis, Fisher test, and chisq test. The final results are based on fold changes, as shown in Table 7. Comparative analysis was performed based on the expression abundance values (RPKM) of the transcripts in different groups (Figure 4). The number of up-regulated genes were 45,399, and the number of down-regulated genes were 15,750 identified in the mammary tissue between CSA_VS_CSB; The number of up-regulated genes were 19,808, and the number of down-regulated genes were 40,673 identified in the mammary tissue between CSA_VS_MF; The number of up-regulated genes were 14,049, and the number of down-regulated genes were 74,111 identified in the mammary tissue between CSB_VS_MF. The order of the number of up-regulated expressed genes from low to high after all transcripts were compared between the three groups was CSB < CSA < MF.

**Table 7:** The number of different genes in the treatments.

	**FC.p (up)^†^**	**FC.n (down)^†^**	**FT.g^†^**	**FT.l^†^**	**CT^†^**
CSA_VS _CSB^‡^	45,399	15,750	691	113	3,400
CSA_VS _MF^‡^	19,808	40,673	75	465	5,423
CSB_VS _MF^‡^	14,049	74,111	128	1,601	4,542

† FC.p, fold. Change. Positive (value≥ 1); FC.n, fold. Change. Negative (value < −1); FT.g, fisher.test. Greater; FT.l, fisher.test. Less; CT, chisq.test.

‡ MF, with hay, corn silage, and alfalfa mixed as roughage; CSA, with only corn stalk as forage and with the same level of proximate nutrients to MF; CSB, with only corn stalk as forage and with the same proportion of roughage to MF.

After comparing the three groups, the classification reaching the most differences in the annotation in GO analysis was membrane. The KEGG pathway analysis showed that the calcium signaling pathway was the most downregulated, and the cancer pathway was the most upregulated in CS2 than MF. Focal adhesion was the most downregulated, and the cancer pathway was the most upregulated in CSA. Compared with CSB, ABC transporters were the most downregulated pathway, focal adhesion was the most upregulated pathway, and cancer was the second pathway in CSA.

### 3.5 Enrichment analysis of DEGs pathways in lipid metabolism

**Figure 5:**
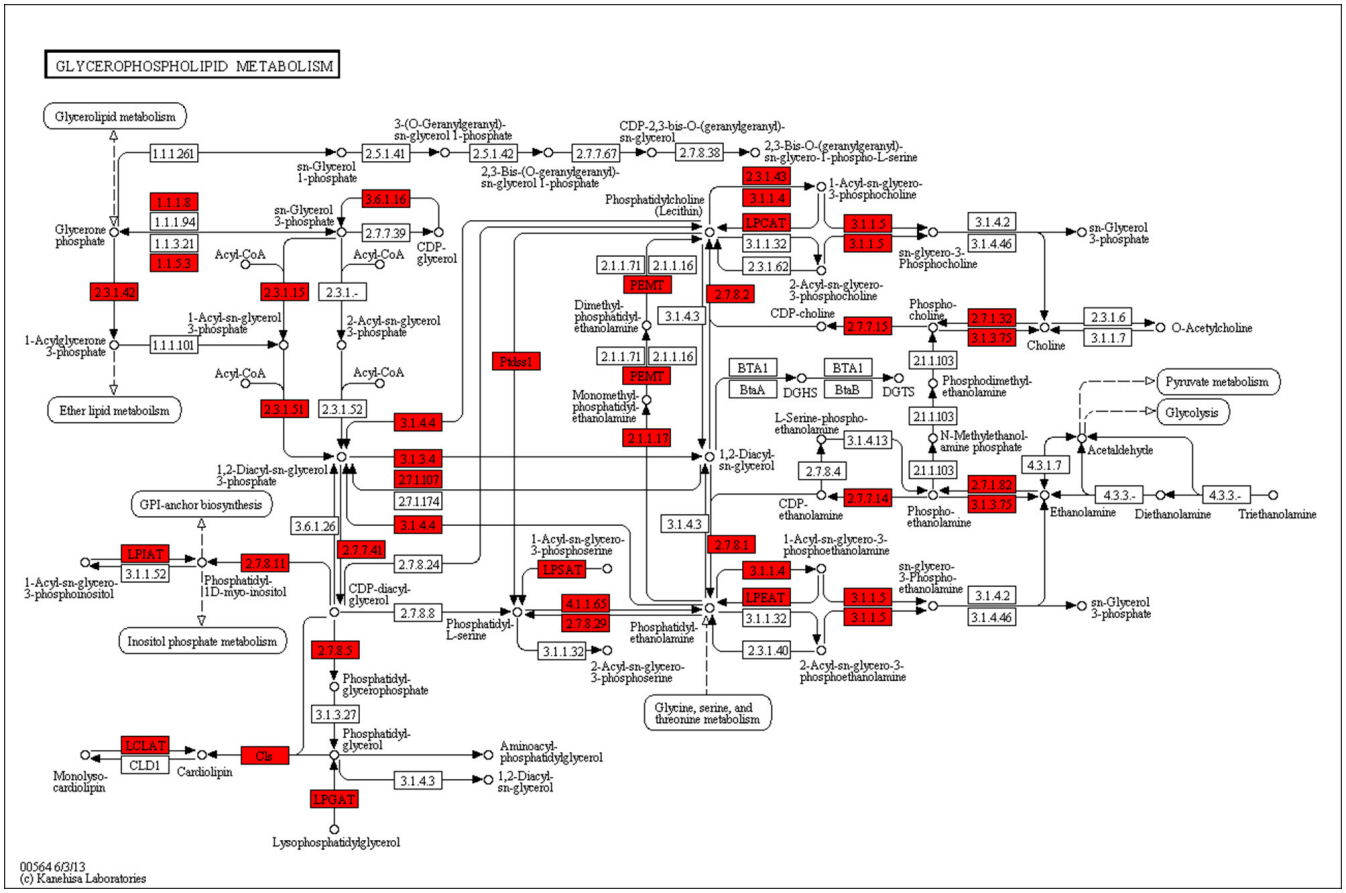
Glycerophospholipid metabolism pathway.

By KEGG analyzing the genes involved in FA synthesis in the mammary glands of dairy cows, 935 transcripts were found to be involved in 16 pathways of lipid metabolism, and 62 genes were found to be significant. DEGs identified in the mammary tissue involved in important lipid metabolism pathways between treatments are shown in Table 8. In the MF group, the differentially expressed genes of the ether lipid metabolism (ko00565), unsaturated FA biosynthesis pathway (ko01040), and FA elongation (ko00062) pathway were significantly lower than those in the CSB group (*P* < 0.05). These differentially expressed genes included *Ethanolamine phosphotransferase (EPT)1, Lysophosphatidylcholine acyltransferase 1 (LPCAT1), peroxisomal FA* β*-oxidation Acyl-CoA Oxidase 1, 3(ACOX1, ACOX 3), Hydroxyacyl-CoA dehydrogenase trifunctional multienzyme complex subunit* β*(HADHB), Fatty Acid Desaturase 2(FADS2), Stearoyl-CoA Desaturase (SCD)*, and *Elongation of Very Long-chain Fatty Acid-like Fatty Acid Elongase 4, 5, 6, 7 (ELOVL4, ELOVL5, ELOVL6, ELOVL7)*. At the same time, the differentially expressed genes of the FA biosynthesis pathway (ko00061) in the MF group were higher than those in the CSB group (*P* < 0.05). These differentially expressed genes included *de novo* synthesis *Acetyl-CoA Carboxylase (ACAC), Fatty Acid Synthase (FASN)*, and *Fatty-acid synthesis II dehydrogenases bacterial 3-ketoacyl-ACP reductase (fabG)*. A map of metabolic pathways involved in differentially expressed genes can be obtained by comparing the differentially expressed genes to the KEGG database. The pathway in which transcripts are most involved in glycerophospholipid metabolism and the most differentially significant genes are produced (Figure 5).

**Table 8:** Differential expressed genes (DEGs) identified in the mammary tissue in main lipid metabolism pathways between treatments.

**Pathway ID**	**Name**	**Treatments^†^**			**FC^‡^**		
		**MF**	**CSA**	**CSB**	**CSA-CSB**	**CSA-MF**	**CSB-MF**
ko00564	AGPAT8	0.71	0.19	0.38	1	1.9	0.90
	AGPAT3_4	0.35	0.19	0.86	1.26	0.88	−1.30
	LPCAT1_2	0.57	0.7	1.64	1.22	−0.29	−1.52
	AGPAT9	0.23	0.73	0.67	−0.12	−1.66	−1.54
	CDS1.CdsA	0.3	0.34	1.1	−0.18	1.69	−1.87
	EPT1	0.19	0.34	0.74	1.12	−0.84	−1.96
	DGK. dgkA	0.12	0.31	0.66	1.09	−1.37	−2.46
	LYPLA3	0.25	0.22	0.66	1.58	0.18	−1.40
	LPGAT1	1.52	1.98	4.26	1.11	−0.38	−1.49
	TAZ	0.15	0.33	0.3	−0.14	−1.13	−1
	psd	0.15	0.29	0.76	1.39	−0.93	−2.34
	PLB1	0.11	0.31	0.8	1.37	−1.49	−2.86
	PLD	0.3	0.36	0.94	1.38	−0.26	−1.65
	LPIN	17.97	5.33	6.02	0.17	1.75	1.58
ko00561	AGPAT8	0.71	0.19	0.38	1	1.9	0.90
	AGPAT3_4	0.35	0.19	0.86	1.26	0.88	−1.30
	LPCAT1_2	0.57	0.7	1.64	1.22	−0.29	−1.52
	LCLAT1	0.71	0.19	0.38	1	1.9	−1.49
	AGPAT9	0.23	0.73	0.67	−0.12	−1.66	−1.54
	DGK. dgkA	0.12	0.31	0.66	1.09	−1.37	−2.46
	LPIN	17.97	5.33	6.02	0.17	1.75	1.58
	DGAT1	0.66	0.66	0.26	−1.34	0	−1.34
	DGAT2	0.16	0.06	0.74	3.62	1.42	−2.21
ko00071	HADHB	7.52	15.67	20.64	0.40	−1.06	−1.46
	CPT1	0.26	0.76	0.67	−0.18	−1.55	−1.37
	ACADL	0.43	0.41	0.94	1.20	0.07	−1.13
	ACSBG	0.71	0.63	0.2	−1.66	0.17	1.83
	ACSL. fadD	0.16	0.4	0.96	1.26	−1.32	−2.58
	ACOX1,3	0.39	0.78	1.01	0.37	−1.00	−1.37
	ACADS. Bcd	0.02	0.1	0.04	−1.32	−2.32	−1
	ACADSB	1.3	1.57	3.14	1.00	−0.27	−1.25
ko00565	LPCAT1	0.57	0.7	1.64	1.22	−0.29	−1.52
	EPT1	0.19	0.34	0.74	1.12	−0.84	−1.96
ko01040	ACOX1,3	0.39	0.78	1.01	0.37	−1.00	−1.37
	FADS2	0.08	0.25	0.51	1.03	−1.64	−2.67
	SCD.desC	0.31	0.28	1.22	2.12	0.15	−1.98
	ELOVL5	0.38	0.99	1.55	0.65	−1.38	−2.03
	ELOVL6	0.32	0.5	1.01	1.01	−0.64	−1.66
	HADHB	7.52	15.67	20.64	0.40	−1.06	−1.46
ko00062	FADS2	0.08	0.25	0.51	1.03	−1.64	−2.67
	ELOVL4	0.27	0.12	0.54	2.17	1.17	−1
	ELOVL5	0.38	0.99	1.55	0.65	−1.38	−2.03
	ELOVL6	0.32	0.5	1.01	1.01	−0.64	−1.66
	ELOVL7	0.33	0.45	0.98	1.12	−0.45	−1.57
ko00061	ACAC	2.76	2.17	1.37	−0.66	0.34	1.01
	FASN	48.16	35.42	26.14	−0.44	0.46	1.07
	fabG	0.91	0.74	0.33	−1.16	0.29	1.46

† MF, with hay, corn silage, and alfalfa mixed as forage; CSA, with only corn stalk as forage and with the same level of proximate nutrients to MF; CSB, with only corn stalk as forage and with the same proportion of forage to MF.

‡ FC: Fold Change of the very significantly DEGs: Fold Change ≥ 1 or < -1 means up-regulation or down-regulation. CSA-CSB means CSA_VS _CSB; CSA-MF means CSA_VS _MF; CSB-MF means CSB_VS _MF.

### 3.6 The high-throughput sequencing results verified using RT-qPCR

**Figure 6:**
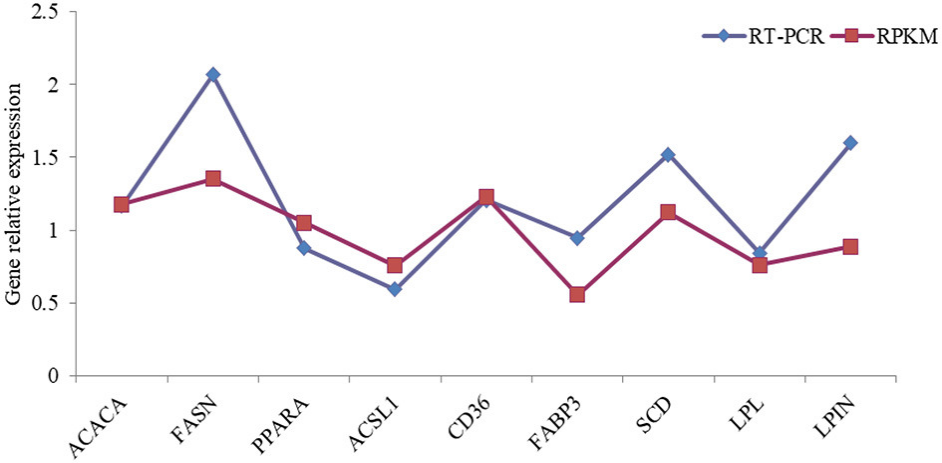
The comparison of high-throughput sequencing and RT-PCR relative expression for fatty acid synthesis-related genes in dairy cows. ACACA, Acetyl-CoA Carboxylase; FASN, Fatty Acid Synthase SCD, Stearoyl-CoA Desaturase; LPL, lipoprotein lipase; PPARA, peroxisome proliferators-activated receptors α; ACSL1, Acyl-CoA Synthetase Long Chain Family Member 1; CD36, luster of differentiation 36; FABP3, Fatty Acid Binding Protein 3; LPIN, Lipid phosphate phosphohydrolase.

The differential expression of nine selected differentially expressed genes, such as *ACACA, SCD, FASN, LPL, PPARA, ACSL1, CD36, FABP3*, and *LPIN*, was validated using RT-qPCR to provide additional evidence for the gene expression profiles recorded at the transcriptome level. The relative expression levels of the selected genes verified using RT-qPCR in a further cohort of samples agreed with the RNA-seq data, although this magnitude varied (Figure 6).

## 4 Discussion

Multiple mechanisms regulate the DMI of ruminants, but the change in dietary starch concentration was ineffective in the DMI of dairy cows (19, 20). Feeding similar NDF content to corn silage-based diets using alfalfa hay, wheat straw, and corn stover can produce similar animal performance. Still, forage with high NDF decreased the milk yield in dairy cows (21). In our study, the CSB diet with higher NDF and lower net energy decreased milk yield compared with the MF and CSA diets fed to dairy cows. As the main forage, the alfalfa hay produced a higher milk yield than the corn stover forage to feed cows. Still, DMI, milk protein, and lactose showed no difference between these treatments (7, 22). A high starch level diet was associated with “Milk fat depression” (8, 23). Still, different dietary starch levels did not affect DMI, milk protein, or lactose in the early lactation of dairy cows and goats (24, 25). Cabrita et al. (26) showed that diets with the same crude protein levels and different starch concentrations resulted in no change in the milk lactose of dairy cows. The milk lactose content could not be manipulated using a diet except under extreme and unusual feeding situations (27). In our study, a single corn stover forage diet (CSA and CSB) decreased milk yield, and a high concentration in the CSA reduced milk fat content of dairy cows was obtained compared to a mixed forage diet.

SMFAs in milk decreased in CSA and CSB, which agreed with Chilliard et al. (28) that cows fed a high dietary C18 FA could decrease medium-chain FA secretion and concentration through dilution with a large amount of LCFA. LCFAs in milk were obtained from arterial blood, and these LCFAs were derived from diets. Our results showed that cows fed with the CSA and CSB diets produced a high amount of UFAs in milk, supporting the hypothesis that cows fed with a high amount of UFA diet produced relatively high proportions of UFAs in milk (29).

The proliferation, differentiation, and survival of normal cells were regulated by interlinked pathways that transmit and integrate signals from growth factors, hormones, and cell–cell and cell–matrix interactions. These pathways can turn into cancer pathways by altering component proto-oncogenes and tumor suppressors. Focal adhesions are dynamic multi-protein complexes that participate in extracellular matrix adhesion and play an important role in translating extracellular matrix stiffness signals into intracellular chemical responses. Focal adhesion dysfunction is critical in tumor cell invasion and metastasis (30). Therefore, the focal adhesion pathway is closely related to the cancer pathway. The activation of MAPK signaling pathways is usually caused by external stimuli such as bacterial infections, inflammatory responses, and oxidative stress (31). Under normal circumstances, the activation of pathways helps regulate immune responses, inflammatory responses, and cellular protective mechanisms. The results indicated cows in CSB group fed diets with low nutrient levels were more involved in the cancer pathway and focal adhesion. In such cases, cows need to use more body mobilization to protect themselves against oxidative stress, inflammation and even more serious diseases. At the same time, the expression of related genes was significantly increased compared to the diet with high nutrient levels.

The ABC family, which is present in all domains of life, is widely acknowledged as one of the largest transporter families (32, 33). ABC transporters have garnered considerable interest due to their capacity to transport diverse substances across cellular membranes and actively facilitate their elimination (34, 35). ABC transport participates in the absorption, accumulation, and excretion of many toxic substances and plays an important role in the defense process of body tissues. The genes of the ABC transport pathway in the high-concentrate corn stover group were more upregulated than those in the low-concentrate corn stover group. This result suggests that a high-concentrate diet may lead to more toxic substances and more genes involved in the defense process. The number of differentially expressed genes in triglyceride metabolism in the MF group was higher than that in the CSA group. This result was consistent with previous results that milk fat content in the MF group was significantly higher than in the CSA group.

Milk fat is the major factor determining the organoleptic quality and the commercial price of milk. LPCAT1 is a key enzyme in the lipid remodeling pathway known as the Lands cycle (36). LPCAT1 may be regulated by the cholesterol biosynthesis pathway (37). In this study, EPT1 and LPCAT1 were higher in the CSB group, showing that milk fat content was lower than in the MF group. Elongation of very long-chain FA (ELOV) proteins is widely present in the genomes of animals, plants, and microorganisms, regulating the length of FA (38). Elovl4 plays a crucial role in the biosynthesis of the so-called “very long-chain (>C24) polyunsaturated fatty acids,” compounds that accumulate in vertebrate photoreceptors that can contain up to C44 (39). In teleosts, most species studied have a single Elovl5 gene that can elongate C18 and C20 PUFA substrates (40). Elovl6 participates in the elongation of SFA and MUFA up to C18, while Elovl7 elongates SFAs and MUFAs of 18–22 carbons (41).

The *FADS2* gene encodes delta-6 desaturase (D6D) and is a member of the FA desaturase gene family. D6D is the key enzyme that catalyzes the transformation of linoleic acid and α-linolenic acid to long-chain polyunsaturated FA (42). *SCD* is the main enzyme involved in monounsaturated FA synthesis in ruminants. A diet with high C18:1c9 inhibited *SCD* mRNA expression in the mammary glands of mice (43). The *SCD* promoter activity was decreased by C18:1c9 in MAC T cells (44). These studies agreed with the current results regarding the changes in the milk FA composition and lipid metabolism pathways. Differentially expressed genes in the CSB diet with high UFA, LCFA, and C18:1c9 are reflected in the dietary FA profile. This may decrease *SCD* and *FADS2* expression and increase *ELOVL4, ELOVL5, ELOVL6*, and *ELOVL7* expressions in mammary glands compared with MF. FabG was identified as an essential gene involved in fatty acid biosynthesis (45). *ACAC* and *FASN* are key enzymes involved in the *de novo* synthesis of FA in mammary tissues (46). High *de novo* FA synthesis in mammary glands increases SMFA content and milk fat (47). The differentially expressed gene results might explain the high amounts of SMFA in the milk fat of cows fed the MF diet.

According to the results of differential gene expression levels of lipid metabolic pathways, the expression levels of genes in the FA biosynthesis pathway in the MF group were significantly higher than in the CSB group. The gene expression of lipid metabolism changed with different diets has been previously described by Zhang et al. (17). The result was consistent with the significantly high milk fat content in the MF and CSB groups. The differentially expressed genes in the FA extension pathway and unsaturated FA synthesis in the MF group were significantly lower than those in CSB, which was consistent with the result that the contents of LCFA and UFA in MF were significantly lower than those in CSB.

## 5 Conclusion

The RNA sequencing data demonstrated that cows fed a low nutritional diet (CSB) had more genes involved in the cancer pathway. Feeding a better nutrition diet (MF) to cows caused the cells to become more active. Cows fed a high-concentrate ratio diet (CSA) had more genes involved in the detoxification defense pathway. According to the analysis of lipid metabolic pathways using KEGG, the DEGs of the FA biosynthesis pathway such as ACAC, FASN and fabG in the MF group were significantly higher than that in the CS group. In contrast, the DEGs of the FA extension pathway and the unsaturated FA synthesis pathway such as ELOVL4, 5, 6, 7 and FADS2, SCD were significantly lower than those in the CS group, consistent with milk fat content and composition results in dairy cows.

## Data Availability

The original contributions presented in the study are included in the article/Supplementary material, further inquiries can be directed to the corresponding author.
